# Guideline adherence and socioeconomic factors in Danish patients referred to secondary care for low back pain: a cross sectional study

**DOI:** 10.1186/s12889-023-16633-4

**Published:** 2023-09-06

**Authors:** Lise Hestbæk, Anne Mette Schmidt, Majbrit Andsbjerg Hald, Nanna Rolving

**Affiliations:** 1 The Chiropractic Knowledge Hub, Campusvej 55, 5230 Odense M, Denmark; 2 Department of Sports Science and Clinical Biomechanics, University of Southern Denmark, Campusvej 55, 5230 Odense M, Denmark; 3 Diagnostic Center, University Research Clinic for Innovative Patient Pathways, Silkeborg Regional Hospital, Falkevej 1-3, 8600 Silkeborg, Denmark; 4 Prehospital Emergency Medical Services, Olof Palmes Allé 34, 8200 Aarhus N, Denmark; 5 Department of Physical and Occupational Therapy, Aarhus University Hospital, Palle Juul-Jensens, Boulevard 99, 8200 Aarhus N, Denmark; 6 Department of Public Health, Aarhus University, Aarhus, Denmark

**Keywords:** Low back pain, Practice guideline, Musculoskeletal manipulation, Exercise therapy, Chiropractic, Physiotherapy, Socioeconomic factors, Health inequity

## Abstract

**Background:**

The pre-referral history of patients with low back pain referred to secondary care is poorly documented, and it is unclear whether it complies with clinical guideline recommendations; specifically, whether they have received appropriate treatment in primary care. This study describes the patient population referred to a spine clinic at a Danish hospital and investigates whether they have received an adequate course of treatment in primary care before referral. Furthermore, a possible association between primary care treatment and socioeconomic factors is estimated.

**Methods:**

We examined self-reported data from 1035 patients with low back pain of at least eight weeks duration referred to secondary care at a medical spine clinic using a cross-sectional design. As an approximation to national clinical guidelines, the definition of an adequate course of treatment in primary care was at least five visits to a physiotherapist or chiropractor prior to referral.

**Results:**

Patients were on average 53 years old, and 56% were women. The average Oswestry Disability Index score was 36, indicating a moderate level of disability. Nearly half of the patients reported pain for over a year, and 75% reported pain below knee level.

Prior to referral, 33% of the patients had not received an adequate course of treatment in primary care. Based on multiple logistic regression with the three socioeconomic variables, age and sex in the model, those who were unemployed had an odds ratio of 2.35 (1.15–4.79) for not receiving appropriate treatment compared to employed patients. Similarly, the odds ratio for patients without vs. with health insurance was 1.71 (1.17–2.50). No significant association was observed with length of education.

**Conclusions:**

Despite national clinical guidelines recommending management for low back pain in primary care, one third of the patients had not received an adequate course of treatment before referral to secondary care. Moreover, the high probability of not having received recommended treatment for patients who were unemployed or lacked health insurance indicates an economic obstacle to adequate care. Therefore, reconsidering the compensation structure for the treatment of back pain patients is imperative to mitigate health inequality within low back pain management.

**Supplementary Information:**

The online version contains supplementary material available at 10.1186/s12889-023-16633-4.

## Background

The largest contributor to the global burden of disease is low back pain (LBP) [1], and in Denmark alone about 900,000 people experience LBP every year [2]. For most patients with LBP, improvement is seen within the first 6–12 weeks, regardless of the treatment received [3, 4]. However, based on studies of clinical course, it has been suggested that the good prognosis might have been overestimated for patients with acute LBP and underestimated the potential for improvement in patients with persistent LBP [5]. Therefore, while it is still important to avoid unnecessary examinations and treatments, it is also crucial to identify and treat those in risk of long-term and disabling LBP. Aside from the impact on the patients’ well-being, this group is also of immense importance from a socio-economic standpoint, as they incur a majority of the costs related to sick leave and health-related pensions [6].

Several countries have developed evidence-based clinical guidelines to optimize patient management and ensure the best possible evidence-based treatment for LBP, with a particular emphasis on preventing long-term problems. Across these international guidelines, a common principle is to provide comprehensive, reassuring information and promote the continuation of everyday activities, including work, to the fullest extent possible. Exercise and manual therapy are recommended when additional treatment is required [3, 7, 8].

To operationalize the national clinical guidelines, regional recommendations for clinical pathways have been published and implemented in Denmark, providing a systematic approach to managing patients with non-specific LBP in both primary and secondary care [9]. The clinical pathways promote the patient-activating strategies recommended in clinical guidelines, including patient information and guidance, encourage patients to remain active, and recommend supervised exercise and/or manual treatment by a physiotherapist or chiropractor. Furthermore, the clinical pathway description provides recommendations for cross-sectoral management, e.g. recommending that if a patient has received appropriate management in primary care for at least eight weeks without improvement, referral for further evaluation in secondary care is recommended [9].

Long-term LBP disproportionately affects individuals with a lower income and those with short or no education [4, 6]. In Denmark, patients must pay 60–82% of the cost for conservative treatments recommended in the clinical guidelines, (e.g. physical therapy or chiropractor) whereas visits to general practitioners and hospitals are free. In addition to monetary expenses, conservative treatments also demand significant personal effort, such as regular exercise or clinic attendance for an extended period. Consequently, there is a considerable risk that those in lower social classes may be less likely to receive the recommended treatment. Given that poorer adherence to treatment recommendations can result in increased morbidity and absenteeism [10], this scenario presents an increased risk of widening the social inequality gap in patients with LBP. Acknowledging such barriers is essential to reduce health inequity due to LBP.

The pathways of patients with LBP before referral to secondary care are poorly described in the scientific literature. Evidence has yet to be produced to highlight whether the treatment of these patients follow the recommendations in national clinical guidelines with respect to primary care management. Accordingly, this study investigates whether they have received an adequate course of treatment in primary care before referral to at specialized hospital-based medical spine clinic, and to what extent adherence to guidelines was related to socioeconomic factors.

## Methods

### Design

A cross-sectional study based on questionnaire data.

### Setting

The Medical Spine Clinic at Silkeborg Regional Hospital is the only specialized medical spine clinic in the Central Denmark Region, a region populated by 1.3 million inhabitants. This means that all patients in the region, who do not show satisfactory improvement from treatment in primary care and have no present indication for surgery, are referred to this clinic (around 5000 patients per year with back and/or neck pain).

### Participants

Following referral, patients were sent an email invitation to a consultation at the Medical Spine Clinic. This included a link to a questionnaire to be completed before arrival, to support the medical history taking during the consultation. All patients who completed the questionnaire electronically from September 1st 2021 to April 21st 2022, and who consented to the use of their data for research and quality development purposes, were included if they indicated LBP (as indicated on an accompanying drawing) as their primary complaint in the questionnaire.

### Variables

Responses to the questionnaire were recorded electronically via SurveyXact version 13 (Rambøll Management Consulting. www.surveyxact.dk), and all included variables were retrieved from these questionnaires.

#### Health-related variables

- BMI: weight in kg/m^2^
- Pain intensity: average pain intensity for the last 14 days measured by the Numerical Rating Scale (0–10)
- Pain duration: 3–6 months; 7–12 months; > 12 months
- Pain below the knee: yes/no
- Comorbidity: number of self-reported diseases in addition to LBP, divided into three categories: 0; 1–2; > 2
- Disability: Oswestry Disability Index (ODI) converted to a percentage score [11]
- Level of physical activity prior to the onset of LBP: elite sports; recreational sports; walking/cycling/other; little to no exercise.

#### Other variables

- Gender: as defined by the Danish civil registration number
- Age: as defined by the Danish civil registration number
- Physiotherapy treatment: '0 times'; '1–4 times'; '5–10 times'; 'more than 10 times’. The last visit had to be within a year of completing the questionnaire.
- Chiropractor treatment: '0 times'; '1–4 times'; '5–10 times'; 'more than 10 times’. The last visit had to be within a year of completing the questionnaire.
- Other treatment: free text
- Education: Educational level was operationalized as the highest completed education and was categorized into three groups according to the International Standard Classification of Education (ISCED) 2011: low (ISCED 0–2: primary/secondary school); medium ((ISCED 3–4: vocational training/short higher education); high (ISCED 5–8: tertiary/higher academic education)[12]
- Job status: divided into four categories: 'unemployed'; 'retired (early retirement, state pension or disability pension)'; 'student/homemaker’; 'employed' (full-time, part-time, or self-employed)
- Sick leave in the past 6 months: yes/no (if yes, number of days)
- Health insurance: Yes; no; don't know.

### Analyses

The relevant regional clinical pathway recommends that in case of no improvement after two weeks, physiotherapeutic or chiropractic treatment should be initiated. Further, that if “*all relevant treatment”* has been attempted in primary care, referral to secondary care can be considered after 8 weeks [9]. Therefore, the criterion for a patient receiving an adequate treatment in the present study was to visit a physiotherapist or a chiropractor. Based on the authors’ clinical experience, a course of four treatments or less is not enough to obtain satisfying results in patients with persistent (> 8 weeks) low back pain. Thus, an adequate course of treatment was defined as reporting to have received at least five sessions with a physiotherapist or chiropractor prior to referral.

Descriptive statistics were utilized for the presentation of the population as a whole and for two groups: those who received an adequate treatment course and those who did not. To evaluate potential differences between these groups, t-tests, chi-square tests, and Mann–Whitney rank sum tests were applied, depending on the variable type and distribution.

Multiple logistic regression was used to determine the association between receiving an adequate course of treatment before referral and the three socioeconomic variables (education, job status and health insurance), and the results are presented as odds ratios (OR). Education, job status, and health insurance were included in the model as independent variables, and the model was further adjusted for age and gender. No imputation was performed and thus all regression analyses were based on complete cases.

Data was processed and analyzed using Stata/IC, version 17 (StataCorp, College Station, Texas, USA) with an applied significance level of 5%.

### Sensitivity analysis

Given that the definition of an adequate course of treatment relies on subjective assessment, we conducted a sensitivity analysis by repeating the regression analyses with an adequate treatment course defined as at least one visit (instead of five) to either a physiotherapist or chiropractor.

The study has been reported according to the STROBE guidelines [13].

## Results

A total of 1051 patients completed the questionnaire, with 1035 responding to questions about pre-referral treatment in primary care. Patients were 53 years old on average, and 56% were women. The average ODI score was 36, indicating a moderate level of disability. Nearly half of all patients had experienced pain for over a year, and 75% reported pain that radiated below the knee. Further details are presented in Table 1.

**Table 1:** Patients with low back pain referred to The Medical Spine Clinic at Silkeborg Regional Hospital. Presented as n (%) if nothing else stated

	*Total population* *N* = *1035*	*Received adequate treatment*^a^ *n* = *692 (67%)*	*Not received adequate treatment*^a^ *n* = *343 (33%)*	*Test for difference*
***Sex**** (n* = *993)*				
*Women*	574 (56)	406 (59)	168 (50)	0.005^c^
***Age,**** years (n* = *993)*				
*mean (SD)*	53.0 (15.0)	51.9 (15.0)	55.3 (14.9)	< 0.001^t^
*min/max*	22—88	22—88	24—87	
***BMI,**** kg/m*^*2*^* (n* = *999)*				
*median (IQR)*	26.6 (23.6–30.4)	26.6 (23.8–30.5)	26.6 (23.5–30.3)	0.761^r^
*min/max*	16.1 – 56.8	16.1 – 56.8	17.2 – 53.4	
***Pain intensity**** past 14 days (n* = *1035)*				
*NRS median (IQR)*	6 (5–8)	6.5 (5–8)	6 (4–8)	0.300^r^
*NRS min/max*	0–10	0–10	0–10	
***Pain duration**** (n* = *1035)*				
< *3 months*	157 (15)	82 (12)	75 (22)	< 0.001^c^
*3–6 months*	223 (22)	134 (19)	89 (26)	
*7–12 months*	174 (17)	122 (18)	52 (15)	
> *12 months*	481 (46)	354 (51)	127 (37)	
***Pain below the knee**** (n* = *917)*				
*Yes, n (%)*	688 (75)	457 (74)	231 (77)	0.278^c^
***Co-morbidity**** (n* = *1035)*				
*0 other diseases*	415 (40)	287 (41)	128 (37)	0.432^c^
*1–2 other diseases*	482 (47)	314 (45)	168 (49)	
> *3 other diseases*	138 (13)	91 (13)	47 (14)	
***Disability**** (ODI) (n* = *1035)*				
*mean (SD)*	36.4 (16.5)	37.0 (16.1)	35.0 (17.3)	0.063^t^
*min/max*	0—89	0—88	0—89	
***Physical activity before LBP**** (n* = *1007)*				
*Elite sports*	31 (3)	22 (3)	9 (3)	< 0.001^c^
*Recreational sports*	395 (39)	285 (42)	110 (33)	
*Walking, cycling, other*	419 (42)	280 (42)	139 (41)	
*Little or none*	162 (16)	84 (13)	78 (23)	
***Highest education**** (n* = *983)*				
*Primary/secondary school*	197 (20)	129 (20)	68 (21)	0.887^c^
*Vocational training/short higher education*	441 (45)	294 (45)	147 (45)	
*Tertiary/higher academic education*	345 (35)	122 (36)	112 (34)	
***Job status**** (n* = *889)*				
*Unemployed*	42 (5)	18 (3)	24 (8)	< 0.001^c^
*Retired*	260 (29)	153(26)	107 (36)	
*Student/homemaker*	54 (6)	38 (6)	16 (5)	
*Employed*	533 (60)	379 (64)	154 (51)	
***Sick leave past 6 months***				
*Yes (n* = *1035)*	174 (17)	124 (18)	50 (15)	0.176^c^
*Number of days (n* = *174)* *median (IQR)*	60 (30–120)	60 (35–120)	42.5 (23–90)	0.007^r^
***Health insurance**** (n* = *1009)*				
*Yes*	331 (33)	248 (37)	83 (25)	< 0.001^c^
*No*	557 (55)	354 (53)	203 (60)	
*Do not know*	121 (12)	70 (10)	51 (15)	

*IQR* Interquartile Range, *NRS* Numerical Rating Scale, *ODI* Oswestry Disability Index

^a^A course of ≥ 5 treatments with a physiotherapist of chiropractor

^t^t-test for normally distributed continuous variables

^c^Chi^2^ test for categorical variables

^r^Mann-Whitney rank sum test for non-normally distributed variables

Prior to referral, 33% of patients had not received an adequate course of treatment in primary care (i.e. less than 5 consultations), while 38% had received a physiotherapeutic treatment course, 11% had a chiropractic treatment course, and 17% had both. Only 57 patients (5.5%) had consulted other care providers, which might have provided manual therapy or exercise therapy (osteopaths: 29, masseurs: 21, craniosacral therapists: 7, and naprapaths: 0).

Patients who received an adequate treatment course were younger, a higher proportion were women, they had experienced pain for a longer duration, reported higher levels of physical activity, were more likely to be employed, and had a higher rate of health insurance, than patients who did not receive adequate course of treatment. Details are shown in Table 1.

Analyses of the relationship between not receiving an adequate treatment course and socioeconomic factors revealed statistically significant associations with employment status and health insurance. The adjusted OR was 2.35 for unemployed patients compared to employed patients. For health insurance, the ORs were 1.71 and 2.18 respectively for patients without health insurance or not knowing their health insurance status, compared to those with health insurance. No significant relationship was observed between adequate course of treatment and the length of education (ORs 1.04 and 1.05, respectively) (see Table 2 for details).

**Table 2:** Odds ratios for not having received an adequate course of treatment prior to referral to The Medical Spine Clinic as a function of socioeconomic variables. OR Odds ratio, CI confidence interval. All three socioeconomic variables included in the model and furthermore adjusted for age and sex

	***Adjusted OR (95%CI)***
***Highest education**** (n* = *983)*	
*Tertiary education*	1 (ref)
*Vocational/short*	1.04 (0.74–1.45)
*Primary/secondary school*	1.05 (0.69–1.61)
***Job status**** (n* = *889)*	
*Employed*	1 (ref)
*Unemployed*	2.35 (1.15–4.79)
*Retired*	1.39 (0.92–2.12)
*Student/homemaker*	0.64 (0.30–1.35)
***Health insurance**** (n* = *1009)*	
*Yes*	1 (ref)
*No*	1.71 (1.17–2.50)
*Don’t Know*	2.76 (1.23- 2.25)

### Sensitivity analysis

When the definition of treatment was revised from requiring at least five visits to requiring only one, 42% of patients had received treatment from a physiotherapist, 8% had received treatment from a chiropractor, and 35% had received treatment from both. Only 15% (*n* = 156) did not meet the treatment criterion, so the estimates in the regression analyses had wider confidence intervals than in the primary analysis. However, the general picture of the relationships remained consistent (see Additional File 1).

## Discussion

The patient population referred to the Medical Spine Clinic were generally characterized by factors indicating a poor prognosis: middle-aged, pain radiating below the knee, relatively high pain intensity, and long duration. All these factors have previously been associated with an increased risk of long-lasting or recurrent LBP [14–17], and sick leave [18].

Despite the apparent severity of symptoms, we found that a third of the referred patients had not received the recommended treatment in primary care before referral to secondary care, although this is recommended in both Danish and international clinical guidelines [3, 7, 8]. This lack of recommended treatment was not related to education, but strongly related to the investigated economic factors (employment and insurance), e.g. those that were unemployed were possibly more than twice as likely to *not* having received an adequate course of treatment before referral. The literature generally indicates that recommendations for treating LBP have remained essentially unchanged over the past 20 years [10, 19]. The common principle across clinical guidelines is to recommend exercise and manual therapy when treatment is required in addition to information and promotion of the continuation of everyday activities [3, 7, 8]. Our findings correspond to an international systematic review that examined whether referral criteria from guidelines for treating LBP were followed in practice and found that 15–20% of general practitioners refer their patients to physiotherapy [20]. Among patients in our study, 38% received at least five treatments from a physiotherapist. This suggests that many general practitioners in Denmark recognize the benefits of referring patients to physiotherapy. However, for some patients, a lack of financial resources, motivation, or other social and personal barriers may hinder the completion of an adequate course of treatment with a physiotherapist.

The cross-sectional nature of the present study does not allow to draw conclusions in relation to causality, as other factors might influence the association between treatment and socioeconomic factors, e.g. less willingness to pay for treatment among the unemployed due to lower motivation. Nevertheless, the significant demonstrated associations between lack of treatment in primary care and both unemployment and lack of health insurance should cause some concern. As private health insurance is often provided by the employer, people in employment more often have health insurance than the unemployed, but our multiple regression model demonstrated that both factors were independently associated with receiving treatment. Although being conducted in just one spine clinic in Denmark, this spine clinic covers an uptake area of app. 1,3 million citizens as it is the only specialized spine clinic in the Central Denmark Region. This means that patients from both rural and urban geographical areas of the Central Denmark Region are referred to this spine clinic. Overall, we consider study findings are generalizable to welfare societies and health care systems similar to the Danish, i.e. Sweden, Norway, the Netherlands, where treatment in general practice and hospitals are tax-financed, and treatment in physiotherapy and chiropractor practice to some extent is reimbursed. At the same time, people on sick leave or unemployed in those countries receive welfare payments to minimize the negative economic impacts on citizens. Most health insurances include coverage of costs for physiotherapy or chiropractic treatment, and thus affects social equality in health negatively, as it is limited to people who can afford a private health insurance, or people employed in companies offering this as part of the contract.

The present study highlights some issues of social inequality in healthcare, even in a country with tax-financed health care and addressing this concern may necessitate a revised payment structure for the current services offered in this field. In societies where provision of health care services is more dependent on the individual's private health insurance, and where sick leave and unemployment results in a minimum of social security benefits, the associations seen in our study may be significantly stronger. An overview article from The Lancet's special issue on LBP in 2018 emphasized the global need for implementing clinical guidelines and changing payment structures to improve treatment options and reduce the use of 'low-value care' [10].

Some respondents opted for paper-based rather than electronic questionnaires. These responses are not included in the database and unfortunately, we were not able to retrieve information about these patients. According to the clinicians, the group of non-responders likely consists of a higher proportion of patients with limited digital literacy, such as the elderly, or ethnic minority groups with poor Danish skills, who also tend to have lower income on average [21], Therefore, our description, as well as the differences between the treated and untreated groups presented in Table 1, may be biased concerning age, education, ethnicity, and job status. However, since the untreated group in our sample is older and less educated, we believe the reported differences would only be larger if the entire patient population from the Spine Center was included. For the same reasons, nonparticipation is unlikely to weaken the estimated relationship between treatment received and socioeconomic status.

Furthermore, basing our definition of ‘adequate course of care’ on self-report of reaching a specific cut point (at least five treatments) can be challenged, both in relation to recall bias and in relation to the choice of cut point. However, even when the threshold was lowered to just one visit to a physiotherapist or chiropractor, the associations to employment status and health insurance remained.

Ideally, we should have included information about the patients' income. Unfortunately, income was not available in this questionnaire. Still, job status is used as a proxy for income, as we believe it is a reasonable assumption that people in employment have a higher income than unemployed individuals. Moreover, we assume that having health insurance increases the likelihood of being able to afford adequate treatment. Therefore, although we lack direct information on income, our results suggest that those with fewer economic resources are the least likely to undergo the recommended conservative treatment.

Finally, it should be noted that the definition of ‘adequate course of treatment’ only relates to quantity, i.e. at least five sessions with a physiotherapist or chiropractor. Only few patients had sought similar treatment from other professions, so this limitation is unlikely to influence the results. However, there is no information available about the treatment received, and thus receiving ‘an adequate course of treatment’ by this definition does not ensure evidence-based treatment. Still, it is evident that if the patients did not visit a physiotherapist or chiropractor, they did not receive treatment, evidence-based or otherwise.

## Conclusion

Despite national clinical guidelines recommending management for low back pain in primary care, one third of the patients had not received an adequate course of treatment before referral to secondary care. Moreover, the high probability of not having received recommended treatment for patients who were unemployed or lacked health insurance indicates an economic obstacle to adequate care, which is independent of education. Therefore, reconsidering the compensation structure for the treatment of back pain patients is imperative to mitigate health inequality within low back pain management.

### Supplementary Information

**Additional file 1. **Odds ratios for not having visited a physiotherapist or chiropractor at least once before referral to The Medical Spine Clinic, Silkeborg Regional Hospital as a function of socioeconomic variables.

## Data Availability

The datasets generated and analysed during the current study are not publicly available due individual privacy but are available in anonymized form from the corresponding author on reasonable request.

## Abbreviations

CI: Confidence Interval
IQR: Inter Quartile Range
LBP: Low Back Pain
NRS: Numerical Rating Scale
ODI: Oswestry Disability Index
OR: Odds Ratio
SD: Standard Deviation
