# Supplementary material for: Guideline adherence and socioeconomic factors in Danish patients referred to secondary care for low back pain: a cross sectional study
Source: BMC Public Health. 2023 Sep 6;23:1733. doi: 10.1186/s12889-023-16633-4 (PMC10481487; doi:10.1186/s12889-023-16633-4)
Supplement: Supplementary file 1 — Additional file 1. Odds ratios for not having visited a physiotherapist or chiropractor at least once before referral to The Medical Spine Clinic, Silkeborg Regional Hospital as a function of socioeconomic variables. [file 12889_2023_16633_MOESM1_ESM.docx]

**Additional file 1: Odds ratios for not having visited a physiotherapist or chiropractor at least once before referral to The Medical Spine Clinic, Silkeborg Regional Hospital as a function of socioeconomic variables.**

|  | **Unadjusted OR (95%CI)** | **Adjusted OR (95%CI)*** |
| --- | --- | --- |
| **Highest education** |  |  |
| Tertiary education | 1 (ref) | 1 (ref) |
| Vocational/short | 1.01 (0.68-1.50) | 0.97 (0.63-1.51) |
| Primary/secondary school | 1.01 (0.62-1.65) | 0.92 (0.53-1.59) |
| **Job status** |  |  |
| Employed | 1 (ref) | 1 (ref) |
| Unemployed | 4.43 (1.47-6.72) | 3.15 (1.47-6.72) |
| Retired | 1.67 (1.11-2.51) | 1.28 (0.74-2.22) |
| Student/homemaker | 1.25 (0.57-2.77) | 0.67 (0.24-1.85) |
| **Health insurance** |  |  |
| Yes | 1 (ref) | 1 (ref) |
| No | 1.97 (2.29-3.01) | 2.07 (1.22-3.53) |
| Don’t Know | 2.31 (1.30-4.11) | 2.72 (1.39-5.32) |
|  |  |  |

OR: Odds ratio, CI: confidence interval

*All three socioeconomic variables included in the model and furthermore adjusted for age and sex.
